# Bioaccessibility and Gut Microbiota Modulation of Phenolics in *Prunus mume* vs. *Fructus mume*

**DOI:** 10.3390/foods14234067

**Published:** 2025-11-27

**Authors:** Qingzhuang Xie, Zhaolun Tan, Bangyan You, Jinxin Luo, Wei Huang, Ruili Yang, Wu Li

**Affiliations:** 1 Guangdong Provincial Key Laboratory of Food Quality and Safety, College of Food Science, South China Agricultural University, Guangzhou 510642, China; xqz126sa@163.com (Q.X.); tanzhaolun2020@163.com (Z.T.); youbg0306@163.com (B.Y.); luojinxin0124@163.com (J.L.); weih007@scau.edu.cn (W.H.); 2 School of Pharmacy and Food Engineering, Wuyi University, Jiangmen 529020, China

**Keywords:** *Fructus mume*, *Prunus mume*, phenolics, in vitro digestion, colonic fermentation, gut microbiota

## Abstract

*Fructus mume* (FM), the processed product of *Prunus mume* (PM), is a traditional Chinese medicine. The release characteristics and bioactivities of phenolics from PM and FM were compared in the present study. In oral and gastric digestion, both the total polyphenols content released and the antioxidant activities of PM were much higher than those of FM, whereas the opposite trend was observed in intestinal digestion and colonic fermentation. Specifically, during colonic fermentation, the total polyphenols content released of FM was 1.43-fold higher than that of PM, with corresponding antioxidant activities (DPPH and ABTS) of FM being 1.41- and 2.91-fold higher, respectively. Twenty-four individual phenolics were found after gastrointestinal digestions with neochlorogenic acid and chlorogenic acid as the predominant ones. During colonic fermentation, a comparatively higher content of cryptochlorogenic acid and benzoic acid was detected in FM, while a higher content of 3-(3,4-dihydroxyphenyl) propionic acid was detected in PM. Notably, FM has a better effect on regulating the gut microbiota composition than PM, as evidenced by a greater enrichment of beneficial bacteria such as *Bifidobacterium* and *Megamonas*, along with a stronger suppression of the pathogenic *Escherichia–Shigella*. These results provided insights into the digestive properties of polyphenols from PM and FM, indicating that processing of PM into FM potentially enhance its health-improving effects on the colon.

## 1. Introduction

*Prunus mume* Sieb. et Zucc. (PM), an important fruit crop, is widely cultivated in east Asia [1]. Due to the extremely sour taste, it is primarily processed into other foods. Among the products of PM, *Fructus mume* (FM) is the main product in China, processed by drying at low temperature (45–55 °C) and then storing in airtight containers until it turns completely black [2]. FM, as a type of homologous medicine and food [3], has been used for thousands of years in traditional Chinese medicine to relieve cough, treat ulceration and improve digestive function [4]. Previous studies have reported that FM alleviates ulcerative colitis through anti-inflammatory and antioxidant effects [5]. In addition, the Wumei pill [6] and Wumei decoction [7], which use FM as the primary ingredient, have also shown good efficacy in treating ulcerative colitis.

PM contains abundant polyphenols, the total content of which was significantly higher than of common fruits such as apple and orange, ranking second among 33 common fruits [8]. Over 20 phenolic compounds have been identified in PM [9,10]. The major phenolics include flavonoids such as procyanidin B1, epicatechin, and catechin, as well as phenolic acids such as caffeic acid and *p*-coumaric acid [11]. Although PM contains abundant phenolic compounds, their bioaccessibility is limited by binding to proteins and fiber in the plant matrix. The acidic environment of the stomach and the enzymes (pepsin, lipase) secreted during upper gastrointestinal digestion help release part of these phenolic compounds. However, the consumption of polyphenols from food may not provide as many benefits as expected due to generally poor gastrointestinal absorption. A previous study reported that approximately 10% of dietary polyphenols are absorbed within the upper gastrointestinal tract [12]. Those polyphenols that are difficult to release and/or absorb directly reach the colon in intact form, where they are metabolized by colonic microbiota [13]. At the same time, the unabsorbed polyphenols can also modify the gut microbiota composition, thereby having a positive impact on colonic health [14]. Therefore, studying the release and catabolism of food polyphenols at different digestion stages is crucial for understanding their diverse biological activity and beneficial effects on human health.

Previous studies have showed that processing can alter the release and biological activities of polyphenols. For example, solid-state fermentation relatively increased phenolic bioaccessibility and antioxidant activities of mulberry leaves [15]. Our previous results found that the content and distribution of individual phenolic compounds in PM were altered by processing [11]. However, the release and catabolism of phenolics during in vitro simulated digestion and colonic fermentation, as well as the antioxidant and gut microbiota modulation effects of PM and FM have not been elucidated and compared. Therefore, the objectives of this study were (1) to identify, quantify and compare the release and catabolism of phenolics from PM and FM during in vitro simulated digestion and colonic fermentation; (2) to assess the antioxidant activity change of the released phenolics from PM and FM during different digestion stage; and (3) to evaluate the effects of PM and FM on gut microbiota structure and composition.

## 2. Materials and Methods

### 2.1. Materials

*Prunus mume* (PM) and FM were obtained from Guangdong Kanghui Group Co., Ltd. (Chaozhou, China). The plum fruit were harvested and processed as our previous study [11]. Briefly, PM fruits (about 18 g a single fruit weight) were harvested at commercial maturity from an orchard in Puning, Guangdong (23°29′ N, 116°117′ E). After harvesting, they were transported to the laboratory under refrigerated conditions (4 °C). According to the 2020 version of the Chinese Pharmacopoeia collection and processing method, the PM (with no mechanical damage, hard texture, thick flesh) were rinsed 2 times with clean tap water before processing. After rinsing thoroughly and drained, PM was kept at a temperature of 45 °C, baked for 3 days, and then smothered (relative humidity at 70–80%) for 3 days until the color turned black to obtain FM. After removing the core of PM and FM, they were stored at −20 °C until subsequent use.

### 2.2. Chemicals

All reference substances, enzyme, Folin–Ciocâlteu reagent, 6-hydroxy-2,5,7,8-tetramethylchromane-2-carboxylic acid (Trolox), 2,2-Diphenyl-1-picrylhydrazyl, 2,2′-azobis-2-methyl-propanimidamide dihydrochloride (DPPH), and 2,2′-azinobis (3-ethylbenzothiazoline-6-sulfonic acid) (ABTS) were purchased from Sigma Aldrich (Shanghai, China). The standard reference materials, including protocatechuate (≥99%), neochlorogenic acid (≥98%), chlorogenic acid (≥98%), cryptochlorogenic acid (≥98%), 4-hydroxybenzoic acid (≥99%), caffeic acid (≥98%), *p*-coumaric acid (≥98%), ferulic acid (≥99%), isoferulic acid (≥97%), procyanidin B1 (≥95%), catechin (≥98%), epicatechin (≥98%), rutin (≥99%), hyperoside (≥97%), isoquercetin (≥90%), quercetin (≥95%), naringenin (≥98%), syringic acid (≥98%), vanillic acid (≥98%), 2-O-Rhamnosylvitexin (≥98%), and kaempferol-3-O-rutinoside (≥98%), were obtained from Sigma Aldrich (Shanghai, China). The other chemicals, such as potassium chloride (KCl) and sodium bicarbonate (NaHCO_3_), were obtained from Kermel Chemical Reagent Co., Ltd. (Tianjin, China).

### 2.3. In Vitro Simulated Gastrointestinal Digestion

In vitro gastrointestinal digestion was performed according to the INFOGEST method [16]. Simulated salivary fluid (SSF), simulated gastric fluid (SGF) and simulated intestinal fluid (SIF) were prepared in advance according to Table S1.

In vitro static digestion was performed following the INFOGEST 2.0 protocol described by Brodkorb et al. (2019) [16]. Briefly, a solution of the plum sample (1 g), SSF (4 mL), *α*-amylase (0.3 mL, 75 U/mL), CaCl_2_ (25 µL, 44.1 g/L) and ultrapure water (fixed the volume to 5 mL) was shaken for 5 min at 37 °C in a constant temperature shaker and protected from light, constituting the oral phase. The samples were collected at 5 min during this stage. Simulated gastric digestion stage was followed by immediate addition of SGF (4 mL), pepsin (0.42 mL, 2000 U/mL), gastric lipase (0.24 mL, 60 U/mL), CaCl_2_ (2.5 µL) and ultrapure water (fixed the volume to 10 mL). The reaction system was maintained under agitation for 2 h at 37 °C avoiding light, and the pH was maintained at 3.0. The samples were collected at 30, 60, 90 and 120 min during this stage. For simulated intestinal digestion, SIF (4 mL), pancreatin (2.5 mL, 100 U/mL), bile salt (1.5 mL, 200 mg/mL), CaCl_2_ (20 µL) and ultrapure water (fixed the volume to 20 mL, pH to 7.0) were added to the simulated gastric digestion system, mixed and incubated at 37 °C in a water bath with shaking at 200 rpm for 2 h avoiding light. Samples were taken out at 30, 60, 90 and 120 min during this stage. All digested samples were then transferred into a centrifugation tube and ultracentrifuged at 10,000× *g* for 25 min at 4 °C, then the supernatants were collected for further analysis. Moreover, blank digestion without freeze-dried plum powder was performed under the same conditions. The digested residues were lyophilized and used for subsequent fecal fermentation.

Bioaccessibility were calculated as follows [15]:

Bioaccessibility (%) = PC_after_/PC_before_ × 100%

where PC_after_ is the content of the polyphenols released after intestinal phase (IP) digestion, and PC_before_ is the content of the polyphenols before digestion. The acronym “PC” is polyphenols content.

### 2.4. In Vitro Fecal Fermentation

Fresh fecal samples were obtained from six healthy adult volunteers (21–25 years, body mass index of 20.0–23.7, 3 females and 3 males) who reported a healthy dietary structure, no intestinal diseases, no intake of polyphenol-rich foods within the previous 2 days, and no antibiotic treatment within 3 months. The six healthy donors were told of the study’s aims and procedures and signed an experimental informed consent from in agreement with the ethics procedures required at the South China Agricultural University (Guangzhou, China). The samples from intestinal digestion were submitted to in vitro colonic fermentation according to previous published method with little modification [17]. Briefly, the fresh fecal samples from volunteers were rapidly diluted in pre-sterilized 10% (*v/v*) PBS buffer solution to obtain 10% (*v*/*v*) fecal slurry to be used as the inoculum for fermentation. Then, 1 mL of the fecal slurry was added into 9 mL of culture medium containing 100 mg of residues obtained after the intestinal digestion step. The basal nutrient medium containing 4.5 g NaCl, 4.5 g KCl, 2.0 g pectin, 4.0 g mucin, 0.69 g MgSO_4_·H_2_O, 1.0 g guar gum, 0.8 g L-cysteine, 0.5 g KH_2_PO_4_, 0.5 g K_2_HPO_4_, 3.0 g casein, 2.0 g arabinogalactan, 1.5 g NaHCO_3_, 0.4 g cholate, 0.005 g FeSO_4_·7H_2_O, 0.08 g CaCl_2_, 1 mL Tween 80 and 4 mL resazurin (0.025%, *w*/*v*) was prepared as described by Di Naso et al. (2011) [18]. Samples were incubated in an anaerobic workstation (80% N_2_, 10% CO_2_ and 10% H_2_) at 37 °C before being sampled at 0, 1, 3, 6, 12 and 24 h. Analogously, groups without digestive residues served as the negative control. After the completion of each stage, the part slurry of each sample was taken out for gut microbiota analysis, while the remaining slurries were centrifuged and stored at −80 °C until subsequent UPLC-Q-Extractive Orbitrap/MS analysis and gut microbiota analysis, respectively.

### 2.5. Determination of Total Phenol Content

The total phenolic content was determined by the Folin–Ciocâlteu method [19]. The sample solution or gallic acid standard solution (25 μL) was added to a 96-well plate, and 125 μL of 0.2 mol/L Folin phenol reagent was mixed well in a shaker and left to react at room temperature for 10 min avoiding light. Then, 125 μL of saturated Na_2_CO_3_ solution was added. After 30 min, the absorbance value was detected by a microplate reader (Molecular Devices, Silicon Valley, CA, USA) at 765 nm, and the results expressed gallic acid equivalent per gram of dry matter (mg GAE/g DW).

### 2.6. Analysis of Phenolic Composition and Content by UPLC-Q-Exactive Orbitrap/MS

The polyphenols and catabolites were extracted from digestion and fecal fermentation samples following a previously published method [15] with some modifications. Briefly, 100 mg of each sample was extracted with 1 mL of methanol and sonicated for 15 min. Then, the mixture was centrifuged for 10 min at 5000 rpm. Next, the supernatant was filtered through a 0.22 μm polytetrafluoroethylene (PTFE) filter and stored at −80 °C until analysis.

The qualitative and quantitative analysis of phenolic extracts was performed by UPLC-Q-Exactive Orbitrap/MS according to our previous study [11]. Liquid phase conditions: an Agilent Poroshell HPH-C18 column (2.1 × 150 mm, 4 μm) was used for the separation of phenolics; the column temperature was set at 40 °C; the mobile phases A and B were ultrapure water (containing 1‰ formic acid) and acetonitrile (containing 1‰ formic acid), respectively; the flow rate was 0.3 mL/min; the injection volume was 5 μL; the gradient elution: 0~3 min 95~85% A, 3~11 min 85~70% A, 11~15 min 70~50% A, 15~21 min 50~10% A, 21~22 min 10~95% A. The mass spectrometry conditions were as follows: negative ion mode, protective gas (N_2_): 30 arb, auxiliary gas (N_2_): 10 arb, capillary voltage 3200 V, capillary temperature 320 °C and scan range 100–1500 *m/z*. Qualitative analysis was performed based on retention times, primary mass spectrometry information, and secondary mass spectrometry information, and quantitative analysis was performed based on standard working curves (Table S2).

### 2.7. Determination of Antioxidant Activity in Vitro

The DPPH free radical scavenging and ABTS free radical scavenging ability were used to evaluate the antioxidant activity of PM and FM in the simulated in vitro digestion process. ABTS free radical scavenging ability was determined by 96-well method [20], and the result was calculated as milligrams Trolox equivalents per gram of sample dry weight (mg TE/g DW). DPPH free radical scavenging ability assay was determined by the method of Velazquez et al. (2003) [21], and the unit was expressed in milligrams of TE per gram of sample dry weight (mg TE/g DW).

### 2.8. Gut Microbiota Composition Analysis

Microbial DNA of fecal samples was extracted using a commercial DNA extraction kit (Omega Biotek, Norcross, GA, USA). The V3−V4 regions of the 16S rRNA gene were amplified by the PCR system (Bio-rad, Hercules, CA, USA) using 338F-806R primers. The sequencing was conducted by Majorbio Bio-Pharm Technology Co., Ltd. (Shanghai, China) using an Illumina MiSeq PE300 platform (Illumina, San Diego, CA, USA). After filtering, the data analysis was performed with QIIME 1.8.0 software [22].

### 2.9. Statistical Analysis

All data were shown as mean ± standard deviation (SD). The significant analysis was carried out using SPSS 24.0 software (SPSS; IBM, Armonk, NY, USA). Normality was determined by the Shapiro–Wilk test. Student’s *t*-test was performed to determine significant differences between two groups for the parameters analyzed. One-way analysis of variance (ANOVA) was performed to determine significant differences among multiple groups for the parameters analyzed. The homogeneity of variances was analyzed using Tukey’s post hoc test that was used to determine significant differences (*p* < 0.05) among the means. The heterogeneity of variances was analyzed using Games–Howell test. When the data did not follow a normal distribution, the Kruskal–Wallis H test was used as a non-parametric test. Differences were considered significant at a *p*-value < 0.05. For the analysis of fecal microbial composition, the Majorbio I-Sanger Cloudonline platform was used to process the 16S sequence data of the microbiome. Operational taxonomic units (OTUs) with 97% similarity cut-off were clustered using UPARSE version 7.0, and chimeric sequences were identified and removed. The taxonomy of each OTU representative sequence was analyzed by RDP Classifier version 2.11 against the 16S rRNA database (e.g., Silva v138) using a confidence threshold of 0.7. Alpha diversity was used for evaluating the species diversity, including Simpson index, Chao1 index, Shannon index. Principal coordinate analysis (PCoA) was used to reveal differences among the gut microflora of the three groups. Significant differences were set at *p* < 0.05.

## 3. Results and Discussion

### 3.1. Impact of Processing on the Release of Total Polyphenols During Different Digestion Stages

**Figure 1:**
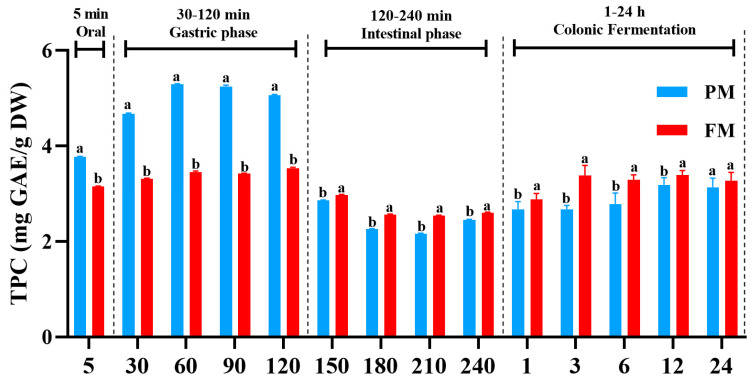
Total phenolic content (TPC) from PM and FM during different digestion stages and colonic fermentation. PM, *Prunus mume*; FM, *Fructus mume*. Data represent mean values ± SD (*n* = 3). For each time point, different letters indicate significant differences (*p* < 0.05).

To evaluate the impacts of digestion in vitro and colonic fermentation on the release of polyphenols from PM and FM, total phenolic content (TPC) was determined, and results were displayed in Figure 1. During oral and gastric digestion (0–120 min), the content of released polyphenols in PM was higher compared with FM, with the highest value of PM (5.29 ± 0.01 mg GAE/g DW) obtained at 60 min, showing a 34.78% increase compared to FM (3.45 ± 0.02 mg GAE/g DW, 60 min). However, the content of released phenolic substances in FM reached 2.60 ± 0.01 mg GAE/g DW and 3.27 ± 0.17 mg GAE/g DW, after intestine digestion and colonic fermentation, respectively, which was significantly higher compared to PM (2.45 ± 0.01 mg GAE/g DW and 3.13 ± 0.19 mg GAE/g DW, respectively). Interestingly, TPC of PM extracts during the gastric phase was higher compared to other digestion phase, which was consistent with results observed in related studies of grapes [23]. Previous studies revealed that gastric conditions increase the releasing of phenolics from the food matrix [24,25], especially molecules with fewer and/or weaker bonds with the food matrix [26]. However, during the gastric phase, compared with PM, the release of phenolic compounds from FM significantly decreased, indicating that processing altered the texture and/or the bonds with the food matrix. The results highlighted the importance of the food state and interaction with other constituents on the release of phenolic compounds. Meanwhile, compared to the gastric phases, the liberation of polyphenols from PM and FM during the intestinal digestion phase (120–240 min) was reduced, which might be attributed to polyphenols being relatively stable in the acidic gastric environment, while they degrade more in the intestine where the pH shifts from acidic to alkaline conditions [27].

During the colonic fermentation stage, TPC showed an overall increasing trend probably because the chemical bonds between the bound polyphenols and food matrix in PM and FM are broken due to microbial action, leading to an increase in polyphenol content [28]. Importantly, during the 24 h in vitro colonic fermentation, FM demonstrated a significantly higher release of polyphenols than PM, with a peak concentration of 3.38 ± 0.21 mg GAE/g DW at the 3 h assessment, possibly due to changes in the food matrix and its bonds with phenolic compounds caused by processing, which resulted in a reduction of released phenolics in gastrointestinal digestion phases and allowed more phenolics attained and released by bacterial cellulases and pectinases in colonic fermentation phase [29]. Collectively, these results indicate that the health effects of FM, particularly concerning colon health, may be more efficient than those of PM.

### 3.2. Impact of Processing on Phenolic Bioavailability

#### 3.2.1. Chemical Characterization of Released Phenolics and Metabolites

A total of 24 individual phenolics and catabolites of PM and FM after exposure to in vitro digestion and colonic fermentation conditions were identified. Details of the UPLC-Q-Exactive Orbitrap/MS characteristics, including the retention time (Rt), chemical structure are summarized in Table 1. There were no significant changes in the 20 compounds obtained from FM after oral and gastrointestinal digestion compared with PM, except for catechin. Catechin from FM was just not released until in colonic stage, inferring that heat treatment might change structure and state of PM so that catechin was not easily released in digestive fluid. Moreover, two monomer phenolic compounds including syringic acid and vanillic acid were detected after gastrointestinal digestion, which was not found in the phenolics extracted from PM and FM using chemical reagents [11] (Table 1 and Figure S1). The possible reason is that syringic acid and vanillic acid existed in bound form, whereas they were degraded or structurally altered due to the intense alkali and acid hydrolysis reaction conditions during the extraction process, such as prolonged hydrolysis, high temperature, strong acid and strong base [30]. Interestingly, syringic acid was detected at each digestion stage, while vanillic acid was not found after the gastric digestion stage (PM) and oral stage (FM), respectively, then detected during colonic fermentation (5.46 and 8.06 mg/100 g DW in PM 3 h and FM 6 h, respectively), suggesting that vanillic acid may exist in both free and bound form, and the bound parts released under the effect of gut microbiota.

Twenty-two phenolic compounds were identified in the extract of PM and FM at the beginning of colonic fermentation stage. Among them, 3-(3,4-dihydroxyphenyl) propionic acid, 2-O-rhamnosylvitexin, kaempferol-3-O-rutinoside and isoferulic acid newly appeared. 3-(3,4-dihydroxyphenyl) propionic acid was identified, which was one of common metabolites in the human circulation generated from gut microbiota catabolism of polyphenols. Isoferulic acid can be generated from caffeic acid via gut microbiota-mediated methylation [31]. In addition, as an isomer of ferulic acid, isoferulic acid can be formed through the bacterial enzyme-mediated migration of a methyl group from one hydroxyl group to an adjacent one on the ferulic acid structure. Kaempferol-3-O-rutinoside and 2-O-rhamnosylvitexin are classified as glycosylated flavonoids. Their presence could be a consequence of selective deglycosylation of precursor flavonoid glycosides, facilitated by the extensive glycoside-hydrolyzing capabilities inherent to the gut microbiota [32]. Remarkably, two monomer phenolic compounds, 2-O-rhamnosylvitexin and kaempferol-3-O-rutinoside, just appeared during in vitro colonic fermentation (Table 1 and Figure S2), which may be due to the fact that polyphenols, especially those with high molecular weight, are hard to release and extract. These results robustly demonstrate that unextractable phenolics such as bound and/or high molecular weight phenolics play an important role on colonic health.

#### 3.2.2. Quantitation of Released Phenolics and Metabolites

Further quantitative measurements of the phenolic compounds from PM and FM during in vitro gastrointestinal digestion and colonic fermentation are presented in Table 2 and Table 3. As shown in Table 2, neochlorogenic acid and caffeic acid were the main phenolic acids in the undigested PM and FM, which accounted for (41.92% and 14.06%) and (48.96% and 12.60%) of the total phenolic content, respectively. In addition, neochlorogenic acid were also the main phenolic acids liberated after in vitro oral digestion. Due to changes in texture or bonds with food matrix caused by processing, the content of the polyphenols released from PM was significantly 1.36 times higher than that from FM during the gastric phase. However, during the intestine and colonic fermentation stage, the released content of phenolics from FM was generally higher than PM, showing a similar trend to the TPC liberation result. Neochlorogenic acid (PM, 300.23 ± 24.17 mg/100 g DW; FM, 231.63 ± 25.03 mg/100 g DW) and 3,4-dihydroxyphenylacetic acid (PM, 323.95 ± 20.39 mg/100 g DW; FM, 205.53 ± 0.46 mg/100 g DW) were dominant phenolics liberated in oral phase. The levels of most individual phenolics generally increased or remained stable during the gastric phase, indicating that the strong interactions between polyphenols and food matrices was further weakened by digestive enzymes and juices in gastric section of the gastrointestinal tract. Acidic stomach environment also contributed to the stability of polyphenols. Nevertheless, our results showed most polyphenols showed a decreasing trend during the intestinal phase, which suggested that some polyphenols were sensitive to soluble oxygen and weakly alkaline environments, leading to their degradation under intestinal fluid conditions. In particular, the levels of some flavanols (such as catechins and epicatechins) declined significantly by 98.09% (FM) and 98.53% (PM) compared with those at gastric stage. The results suggest that flavanols were sensitive to soluble oxygen and weakly alkaline environments, leading to their degradation under intestinal fluid conditions [33]. Furthermore, naringenin had the highest bioaccessibility during intestinal digestive stage, followed by hyperoside. It is worth noting that the bioaccessibility of naringenin in PM during the intestinal digestion stage was 3.69 times higher than that in FM (Table 2). The result indicated that processing could affect the bioaccessibility of phenolics.

For the colonic fermentation phase (Table 3), except for a steady increase in cryptochlorogenic acid and 3-(3,4-Dihydroxyphenyl) propionic acid, the contents of other phenolic compounds reached their peaks at certain points and then decreased. Particularly, caffeic acid and *p*-coumaric acid were reported as the main bound polyphenols in PM and FM [11], showing an increasing trend from 1 to 6 h of fermentation and then decreasing after 6 h. This indicates that bound phenolics are hardly released during the gastrointestinal stage and then are attained in colon. However, isoferulic acid and ferulic acid showed an upward and then stable trend, suggesting that caffeic acid and *p*-coumaric acid might undergo methylation to produce ferulic acid and isoferulic acid [34]. Moreover, chlorogenic acids (neochlorogenic acid, chlorogenic acid and cryptochlorogenic acid) were detected in fermented extracts and accumulated over time. The reason may be that the bound chlorogenic acids were converted to the free chlorogenic acid by the gut microbiota and released into the fermentation broth. Overall, the content of most of the individual phenolic compounds from FM in the colonic fermentation was significantly higher than that of PM, indicating that processing can effectively increase the release of plum phenolics in the colon and may be more conducive to their utilization by the human body especially considering the low bioavailability of polyphenols in upper gastrointestinal tract.

### 3.3. Impact of Processing on Antioxidant Activity Change During Different Digestion Stages

**Figure 2:**
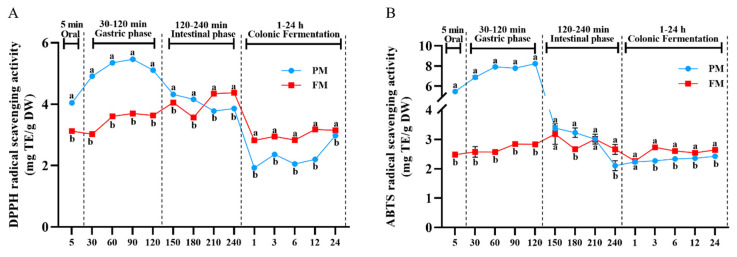
Effect of simulated digestion on DPPH (**A**) and ABTS (**B**) radical scavenging activity in vitro gastrointestinal digestion and colonic fermentation. PM, *Prunus mume*; FM, *Fructus mume*. Data represent mean values ± SD (*n* = 3). For each time point, different letters indicate significant differences (*p* < 0.05).

DPPH and ABTS assays were carried out to assess the antioxidant capacities of released phenolics during different digestion and colonic fermentation stage (Figure 2A,B). Similar to the results of TPC, DPPH and ABTS radical scavenging capacity of PM gradually increased during gastric digestion phase, reaching the maximum values of 5.46 ± 0.02 mg TE/g DW (90 min) and 8.23 ± 0.06 mg TE/g DW (120 min), respectively, which were significantly higher than those of FM (4.05 ± 0.01 mg TE/g DW and 3.18 ± 0.35 mg TE/g DW, respectively) at 150 min. However, the values of DPPH and ABTS decreased during the small intestine digestion, with the antioxidant activity of PM being lower by 13.50% and 30.23%, respectively, compared to FM. Different trends in antioxidant capacities were observed, which could be attributed to the diverse compositions of polyphenols from PM and FM. As mentioned earlier, the content of catechins and epicatechins in PM were more prominent during the gastric phase, and previous research has confirmed that these compounds readily interacted with salivary proteins during oral digestion, making them less likely to be released into the digestive juices [35]. This suggests that they were released from the flavanol polymer by the action of gastric juice and proteases. In contrast, the DPPH radical scavenging capacity of FM was higher than the DPPH in PM during the intestinal phase (210–240 min), indicating that the polyphenols from FM were more easily released in alkaline intestinal fluid environment. Additionally, the content of *p*-coumaric acid and naringenin from FM was increased from the gastric phase (0.99 ± 0.08 mg/100 g DW and 5.60 ± 0.27 mg/100 g DW, respectively) to the intestinal phase (10.89 ± 0.61 mg/100 g DW and 9.41 ± 0.20 mg/100 g DW, respectively). Therefore, the scavenging ability of DPPH might be affected by the high content of *p*-coumaric acid and naringenin. In addition, the antioxidant capacities of released phenolics from FM increased at 3 h colonic fermentation, similar to the results of TPC, possibly associated with the increase in benzoic acid, chlorogenic acid and neochlorogenic acid.

### 3.4. Impact of Processing on Gut Microbiota Regulation Activity

**Figure 3:**
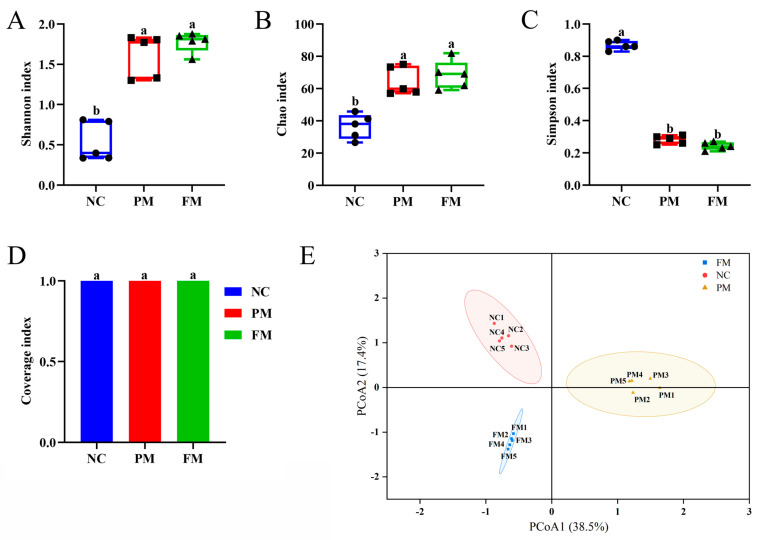
Overall structural changes in gut microbiota after 24 h of colonic fermentation in *Prunus mume* (PM) group, *Fructus mume* (FM) group and negative control (NC; only feces fermentation) group. Shannon index (**A**), Chao1 index (**B**) and Simpson index (**C**) were used as estimators of alpha diversity of gut microbiota. Coverage index (**D**) was used to verify reasonability of sequencing data. Principal coordinate analysis (PCoA) (**E**) was based on relative abundance of OTUs of all samples. Means with no letter (a, b) in common are significantly different (*p* < 0.05).

To further analyze the changes of fecal microbiota community structure from PM and FM, we measured the bacterial composition of the samples collected at 24 of colonic fermentation via high-throughput sequencing of the bacterial 16S rRNA gene. Alpha diversity reflected the richness and diversity of intestinal flora. Good’s coverages index was used to characterize the species coverage of the community during 16S rRNA gene sequencing [36]. As shown in Figure 3, compared to the negative control (NC) group, the Shannon index (Figure 3A) and Chao index (Figure 3B) of PM and FM increased, while the Simpson index (Figure 3C) decreased (*p* < 0.05). The Good’s coverages index (Figure 3D) of all samples in each group was greater than 0.999, indicating that the sequencing data was reliable. These results suggested that both PM and FM had a certain effect on the diversity of the gut microbiota compared to NC group, although the diversity difference between the PM and FM groups was not statistically significant. Beta diversity between different groups was analyzed by principal coordinate analysis (PCoA) [37]. The PCoA score plot showed that the three groups had distinct bacterial communities (Figure 3E).

**Figure 4:**
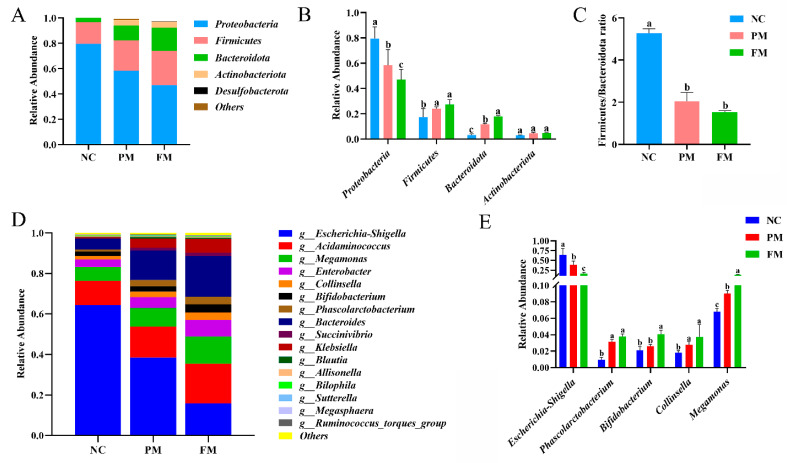
Comparisons of microbiota composition after 24 h of in vitro colonic fermentation in PM, FM and negative control (NC; only feces fermentation) groups. (**A**,**B**) Relative abundance of microbiota at phylum level. (**C**) Ratio of *Firmicutes*/*Bacteroidetes* (F/B). (**D**,**E**) Relative abundance of microbiota at genus level. Data represent mean values ± SD (*n* = 5). Bars with no letter (a, b, c) in common are significantly different (*p* < 0.05) among different groups.

To evaluate the effects of processing on specific microbiota composition, bacterial taxa in all groups were analyzed at the phylum and genus level. At the phylum level, *Proteobacteria*, *Firmicutes*, *Actinobacteria*, and *Bacteroidetes* accounted for the majority of total microbiota in all groups (Figure 4A,B). The *Firmicutes*/*Bacteroidetes* (F/B) ratio is considered to have an important influence in maintaining intestinal homeostasis, and is associated with obesity and inflammatory bowel disease [38]. Compared with NC group, F/B values in the PM and FM groups were significantly decreased by 61.10% and 71.16% (Figure 4C), respectively, during in vitro colonic fermentation, indicating that PM and FM had potential effects in reducing weight and improving inflammatory responses and ulcers.

The relative abundance of the dominant microbiota at the genus level is shown in Figure 4D. The RA of *Escherichia–Shigella* in PM and FM significantly decreased by 39.95% and 75.28% compared with NC group, respectively (Figure 4E). This reduction could be attributed to higher chlorogenic acid released from FM than PM. It was consistent with the results of mung bean coat [39], among which the abundance of *Escherichia–Shigella* was found to be significantly negatively correlated with chlorogenic acid [40]. Notably, the relative abundance of *Megamonas* (increased by 33.13% and 95.26% in PM and FM group, respectively)*, Collinsella* (increased by 51.62% and 104.93% in PM and FM group, respectively) and *Phascolarctobacterium* (increased by 216.66% and 280.64% in PM and FM group, respectively) significantly increased (*p* < 0.05). These bacteria were reported to be beneficial to keep colon healthy. *Megamonas* was found to be negatively associated with the increased risk of colorectal polyps [41]. *Collinsella*, an important bacterium in gut microbiota development, has been linked to a low risk of colon cancer and irritable bowel syndrome [42]. *Phascolarctobacterium* can produce short-chain fatty acids, including acetate and propionate [43,44]. Furthermore, compared with NC group, an increase in relative abundance for *Bifidobacterium* in both PM (23.41%) and FM (92.78%) was observed. *Bifidobacterium* plays a role in boosting overall immunity, including reducing and treating gastrointestinal infections, as well as improving diarrhea, constipation and eczema [45,46,47,48]. Consequently, PM, and especially FM, had potential effects in optimizing the intestinal microbiota structure, which might be the reason that FM has been used as a folk medication for gastrointestinal disorders, e.g., diarrhea, stomach ache and ulceration [49].

**Table 1:** Identification of polyphenols and their metabolites of PM and FM during in vitro digestion and colonic fermentation.

Peak.	RT (min)	Phenolics	Formula	Ion Peak (*m/z*)	Fragment (*m/z*) [Relative Abundance, %]	Source	References
1	4.94	Protocatechuic acid	C_7_H_6_O_4_	153.01839	109.02888 [100]	PM: IP, F1, F3, F6, F12, F24 FM: OP, GP, IP, F1, F3, F6, F12, F24	[50]
2	5.22	Neochlorogenic acid	C_16_H_18_O_9_	353.08844	191.05551 [91], 179.03433 [68], 135.04401 [12]	PM: OP, GP, IP, F1, F3, F6, F12, F24 FM: OP, GP, IP, F1, F3, F6, F12, F24	[51]
3	5.53	Procyanidin B1	C_30_H_26_O_12_	577.13623	289.07211 [100], 407.07773 [64], 125.02317 [75], 425.08859 [40], 451.10410 [10]	PM: OP, GP, IP FM: IP	[52]
4	6.14	Chlorogenic acid	C_16_H_18_O_9_	353.08823	191.05548 [100]	PM: OP, GP, IP, F1, F3, F6, F12, F24 FM: OP, GP, IP, F1, F3, F6, F12, F24	[53]
5	6.21	Catechin	C_15_H_14_O_6_	289.07220	245.08144 [30]	PM: OP, GP, IP, F1, F3, F6, F12, F24 FM: F1, F3, F6, F12, F24	[39]
6	6.38	3-(3,4-Dihydroxyphenyl) propionic acid	C_9_H_10_O_4_	181.04991	137.05969 [100]	PM: F3, F6, F12, F24 FM: F12, F24	[54]
7	6.49	Cryptochlorogenic acid	C_16_H_18_O_9_	353.0833	173.04472 [100], 179.03421 [67], 191.05536 [31], 135.04398 [15]	PM: GP, IP, F1, F3, F6, F12, F24 FM: OP, GP, IP, F1, F3, F6, F12, F24	[51]
8	6.45	*p*-Hydroxybenzoic acid	C_7_H_5_O_3_	137.02339	93.03323 [100]	PM: GP, IP, F1, F3, F6, F12, F24 FM: OP, GP, IP, F1, F3, F6, F12, F24	[55]
9	6.57	3,4-Dihydroxyphenylacetic acid	C_8_H_8_O_4_	167.03420	123.04397 [54]	PM: OP, GP, IP, F1, F3, F6, F12, F24 FM: OP, GP, IP, F1, F3, F6, F12, F24	[56]
10	7.08	Epicatechin	C_15_H_14_O_6_	289.07224	245.08212 [30], 125.02299 [9]	PM: OP, GP, IP, F1, F3, F6, F12, F24 FM: OP, GP, IP, F1, F3, F6, F12, F24	[15]
11	7.12	Caffeic acid	C_9_H_8_O_4_	179.03423	135.04404 [100]	PM: IP, F1, F3, F6, F12, F24 FM: OP, GP, IP, F1, F3, F6, F12	[15]
12	7.22	Syringic acid	C_9_H_10_O_5_	197.04510	182.02152 [100], 153.05495 [14]	PM: OP, GP, IP, F1, F3, F6, F12, F24 FM: OP, GP, IP, F1, F3, F6, F12, F24	[39]
13	7.27	Vanillic acid	C_8_H_8_O_4_	167.03427	152.01057 [100]	PM: GP, F1, F3, F6, F12, F24 FM: OP, F1, F3, F6, F12, F24	[39]
14	8.05	Benzoic acid	C_7_H_6_O_2_	121.02822	77.03805 [11]	PM: IP, F1, F3, F6, F12, F24 FM: OP, GP, IP, F1, F3, F6, F12, F24	[39]
15	8.73	2-O-Rhamnosylvitexin	C_27_H_30_O_14_	577.15686	413.09126 [48]; 293.04826 [69]	PM: F1, F3, F6, F12, F24 FM: F1, F3, F6, F12, F24	[57]
16	8.84	Rutin	C_27_H_30_O_16_	609.14771	300.02783 [100]	PM: OP, GP, IP, F1, F3, F6, F12, F24 FM: OP, GP, IP, F1, F3, F6, F12, F24	[53]
17	8.93	*p*-Coumaric acid	C_9_H_8_O_3_	163.03915	119.04906 [100]	PM: IP, F1, F3, F6, F12 FM: OP, GP, IP, F1, F3, F6, F12	[53]
18	9.19	Hyperoside	C_21_H_20_O_12_	463.08923	300.02783 [58], 301.03546 [34]	PM: OP, GP, IP, F1, F3, F6, F12, F24 FM: OP, GP, IP, F1, F3, F6, F12, F24	[58]
19	9.36	Isoquercetin	C_21_H_20_O_12_	463.08932	300.02779 [100]	PM: OP, GP, IP, F1, F3, F6, F12, F24 FM: OP, GP, IP, F1, F3, F6, F12, F24	[59]
20	9.57	Ferulic acid	C_10_H_10_O_4_	193.04988	178.02635 [90], 134.03616 [100], 149.05968 [35]	PM: IP, F1, F3, F6, F12, F24 FM: OP, GP, IP, F1, F3, F6, F12, F24	[15]
21	9.66	Isoferulic acid	C_10_H_10_O_4_	193.04984	178.02621 [100], 134.03612 [65]	PM: F1, F3, F6, F12, F24 FM: F1, F3, F6, F12, F24	[60]
22	9.87	Kaempferol-3-O-rutinoside	C_27_H_30_O_15_	593.15247	285.04080 [62]	PM: F1, F3, F6, F12, F24 FM: F1, F3, F6, F12, F24	[61]
23	14.63	Quercetin	C_15_H_10_O_7_	301.03583	151.00272 [21], 178.99788 [15]	PM: GP, F1, F3, F6, F12, F24 FM: OP, GP, IP, F1, F3, F6, F12, F24	[15]
24	16.17	Naringenin	C_15_H_12_O_5_	271.06155	151.00255 [43], 119.04892 [14], 177.01808 [6]	PM: IP, F1, F3, F6, F12, F24 FM: OP, GP, IP, F1, F3, F6, F12, F24	[15]

PM, *Prunus mume*; FM, *Fructus mume*; OP, oral phase; GP, gastric phase; IP, intestinal phase. Fermentation broth in different times: F1, 1 h; F3, 3 h; F6, 6 h; F12, 12 h; F24, 24 h.

**Table 2:** Quantitative results and bioaccessibility of released phenolics and metabolites from PM and FM during in vitro digestion (mg/100 g DW).

NO.	Phenolics	Sample	OP	GP	IP (PC_after_)	PC_before_	Intestinal (Bioaccessibility)
1	Protocatechuic acid	PM	Nd	Nd	0.04 ± 0.03 ^a^	0.67 ± 0.04 ^a^	5.97% ^a^
		FM	0.08 ± 0.02 ^B^	0.08 ± 0.01 ^B^	0.24 ± 0.04 ^bA^	2.84 ± 0.07 ^b^	8.45% ^b^
2	Neochlorogenic acid	PM	300.23 ± 24.17 ^aA^	325.68 ± 13.74 ^aA^	22.35 ± 0.42 ^aB^	200.91 ± 42.38 ^a^	11.12% ^a^
		FM	231.63 ± 25.03 ^bA^	220.22 ± 1.40 ^bA^	12.57 ± 1.17 ^bB^	195.34 ± 6.25 ^a^	6.43% ^b^
3	Procyanidin B1	PM	2.79 ± 0.05 ^B^	3.88 ± 0.12 ^A^	0.03 ± 0.00 ^aC^	11.44 ± 1.38 ^a^	0.26% ^a^
		FM	Nd	Nd	0.06 ± 0.00 ^b^	2.92 ± 0.15 ^b^	2.05% ^b^
4	Chlorogenic acid	PM	17.94 ± 0.34 ^aA^	2.48 ± 0.39 ^aB^	2.80 ± 0.10 ^aB^	18.25 ± 1.67 ^a^	15.34% ^a^
		FM	14.66 ± 0.67 ^bB^	23.23 ± 0.78 ^bA^	2.22 ± 0.09 ^aC^	14.57 ± 0.55 ^b^	15.24% ^a^
5	Catechin	PM	9.18 ± 0.16 ^B^	12.58 ± 0.38 ^A^	0.24 ± 0.05 ^C^	34.60 ± 2.12 ^a^	0.69%
		FM	Nd	Nd	Nd	12.99 ± 1.00 ^b^	Nd
6	3-(3,4-Dihydroxyphenyl) propionic acid	PM	Nd	Nd	Nd	Nd	Nd
		FM	Nd	Nd	Nd	Nd	Nd
7	Cryptochlorogenic acid	PM	Nd	0.23 ± 0.09 ^aA^	0.41 ± 0.00 ^aA^	2.92 ± 0.39 ^a^	14.04% ^a^
		FM	5.12 ± 0.20 ^A^	5.01 ± 0.18 ^bA^	0.17 ± 0.02 ^bB^	22.60 ± 0.46 ^b^	0.75% ^b^
8	*p*-Hydroxybenzoic acid	PM	Nd	0.02 ± 0.00 ^aB^	1.39 ± 0.01 ^aA^	6.79 ± 0.23 ^a^	20.47% ^a^
		FM	1.31 ± 0.06 ^A^	1.22 ± 0.02 ^bA^	2.50 ± 0.04 ^bA^	13.69 ± 0.74 ^b^	18.26% ^b^
9	3,4-Dihydroxyphenylacetic acid	PM	323.95 ± 20.39 ^aB^	535.14 ± 17.56 ^aA^	250.13 ± 48.81 ^aB^	Nd	Nd
		FM	205.53 ± 0.46 ^bB^	257.18 ± 8.29 ^bA^	111.18 ± 3.92 ^bAB^	Nd	Nd
10	Epicatechin	PM	45.76 ± 1.60 ^aB^	66.86 ± 1.92 ^aA^	0.98 ± 0.12 ^aC^	45.09 ± 2.12 ^a^	2.17% ^a^
		FM	0.63 ± 0.00 ^bB^	0.88 ± 0.02 ^bA^	0.45 ± 0.03 ^bC^	4.90 ± 0.38 ^b^	9.18% ^b^
11	Caffeic acid	PM	Nd	Nd	0.61 ± 0.10 ^a^	67.40 ± 5.26 ^a^	0.90% ^a^
		FM	0.68 ± 0.04 ^B^	0.72 ± 0.03 ^B^	3.25 ± 0.08 ^bA^	50.26 ± 4.59 ^b^	6.47% ^b^
12	Syringic acid	PM	0.07 ± 0.02 ^aAB^	0.10 ± 0.01 ^aB^	0.36 ± 0.02 ^aA^	Nd	Nd
		FM	0.35 ± 0.06 ^bB^	0.38 ± 0.01 ^bB^	0.47 ± 0.01 ^bA^	Nd	Nd
13	Vanillic acid	PM	Nd	0.38 ± 0.05	Nd	Nd	Nd
		FM	0.21 ± 0.00	Nd	Nd	Nd	Nd
14	Benzoic acid	PM	Nd	Nd	53.74 ± 54.05 ^a^	Nd	Nd
		FM	147.53 ± 6.19 ^bB^	179.20 ± 3.02 ^bA^	98.10 ± 1.54 ^bC^	Nd	Nd
15	2-O-Rhamnosylvitexin	PM	Nd	Nd	Nd	Nd	Nd
		FM	Nd	Nd	Nd	Nd	Nd
16	Rutin	PM	1.91 ± 0.13 ^aA^	2.33 ± 0.04 ^aA^	2.56 ± 0.34 ^aA^	4.34 ± 0.97 ^a^	58.99% ^a^
		FM	2.95 ± 0.06 ^bA^	3.28 ± 0.00 ^bA^	2.64 ± 0.23 ^aA^	4.18 ± 0.39 ^a^	63.16% ^a^
17	*p*-Coumaric acid	PM	Nd	Nd	3.88 ± 0.66 ^a^	33.93 ± 3.95 ^a^	11.44% ^a^
		FM	0.92 ± 0.08 ^B^	0.99 ± 0.08 ^B^	10.89 ± 0.61 ^bA^	28.40 ± 3.88 ^b^	38.34% ^b^
18	Hyperoside	PM	1.10 ± 0.09 ^aA^	1.59 ± 0.12 ^aA^	1.33 ± 0.13 ^aA^	0.84 ± 0.11 ^a^	158.33% ^a^
		FM	1.42 ± 0.00 ^bA^	1.45 ± 0.05 ^aA^	1.28 ± 0.02 ^aB^	0.91 ± 0.44 ^a^	140.66% ^a^
19	Isoquercetin	PM	0.84 ± 0.07 ^aA^	1.21 ± 0.08 ^aA^	1.02 ± 0.08 ^aA^	1.90 ± 0.32 ^a^	53.68% ^a^
		FM	1.08 ± 0.00 ^bA^	1.10 ± 0.04 ^aA^	0.98 ± 0.00 ^aB^	1.14 ± 0.37 ^b^	85.96% ^a^
20	Ferulic acid	PM	Nd	Nd	0.20 ± 0.01 ^a^	6.97 ± 0.80 ^a^	2.87% ^a^
		FM	0.12 ± 0.02 ^A^	0.11 ± 0.01 ^A^	0.67 ± 0.04 ^bA^	6.47 ± 0.74 ^a^	10.36% ^b^
21	Isoferulic acid	PM	Nd	Nd	Nd	40.72 ± 4.77 ^a^	Nd
		FM	Nd	Nd	Nd	26.86 ± 2.34 ^b^	Nd
22	Kaempferol-3-O-rutinoside	PM	Nd	Nd	Nd	Nd	Nd
		FM	Nd	Nd	Nd	Nd	Nd
23	Quercetin	PM	Nd	0.04 ± 0.01 ^a^	Nd	0.18 ± 0.06 ^a^	Nd
		FM	0.18 ± 0.02 ^B^	0.36 ± 0.03 ^bA^	0.12 ± 0.00 ^C^	0.48 ± 0.06 ^b^	25.00%
24	Naringenin	PM	Nd	Nd	7.71 ± 0.33 ^a^	2.31 ± 0.20 ^a^	333.77% ^a^
		FM	4.41 ± 1.00 ^B^	5.60 ± 0.27 ^B^	9.41 ± 0.20 ^bA^	10.39 ± 0.39 ^b^	90.57% ^b^

Nd, not detected; PM, *Prunus mume*; FM, *Fructus mume*; OP, oral phase; GP, gastric phase; IP, intestinal phase; PC_before_, content of polyphenols before digestion; PC_after_, content of polyphenols released after intestinal phase digestion; Bioaccessibility (%) = PC_after_/PC_before_ × 100%. 3-(3,4-dihydroxyphenyl) propionic acid, 3,4-dihydroxyphenylacetic acid and benzoic acid were quantified in *p*-hydroxybenzoic acid equivalents. Data represent mean ± standard deviation (*n* = 3). Different lowercase letters represent a significant difference between PM and FM at same phase (*p* < 0.05). Different capital letters represent a significant difference at different phases (*p* < 0.05).

**Table 3:** Quantitative results of released phenolics and metabolites from PM and FM during colonic fermentation (mg/100 g DW).

NO.	Phenolics	Sample	F1	F3	F6	F12	F24
1	Protocatechuic acid	PM	0.13 ± 0.01 ^aC^	0.15 ± 0.00 ^aC^	0.18 ± 0.02 ^aB^	0.24 ± 0.01 ^aA^	0.01 ± 0.00 ^aD^
		FM	0.49 ± 0.04 ^bB^	0.65 ± 0.06 ^bA^	0.70 ± 0.07 ^bA^	0.46 ± 0.05 ^bB^	0.04 ± 0.01 ^bC^
2	Neochlorogenic acid	PM	215.69 ± 22.41 ^aA^	186.65 ± 17.14 ^aA^	202.25 ± 16.86 ^aA^	210.26 ± 6.87 ^aA^	180.12 ± 11.52 ^aA^
		FM	209.95 ± 19.78 ^aB^	270.42 ± 17.58 ^bA^	188.76 ± 20.15 ^aC^	244.86 ± 24.17 ^aAB^	254.70 ± 15.23 ^bAB^
3	Procyanidin B1	PM	Nd	Nd	Nd	Nd	Nd
		FM	Nd	Nd	Nd	Nd	Nd
4	Chlorogenic acid	PM	44.31 ± 4.26 ^aB^	39.88 ± 3.70 ^aC^	53.33 ± 5.89 ^aAB^	62.17 ± 6.17 ^aA^	54.14 ± 5.37 ^aAB^
		FM	68.15 ± 5.05 ^bB^	94.00 ± 9.27 ^bA^	67.94 ± 5.29 ^bB^	90.07 ± 8.34 ^bA^	95.48 ± 8.90 ^bA^
5	Catechin	PM	0.58 ± 0.07 ^aB^	0.16 ± 0.01 ^aD^	0.30 ± 0.02 ^aC^	0.21 ± 0.01 ^aCD^	0.78 ± 0.05 ^aA^
		FM	0.09 ± 0.01 ^bB^	0.08 ± 0.01 ^bB^	0.15 ± 0.03 ^bA^	0.04 ± 0.00 ^bC^	0.02 ± 0.00 ^bC^
6	3-(3,4-Dihydroxyphenyl) propionic acid	PM	Nd	19.93 ± 1.29 ^D^	32.35 ± 1.55 ^C^	75.20 ± 0.81 ^aB^	191.25 ± 11.83 ^aA^
		FM	Nd	Nd	Nd	17.50 ± 6.61 ^bB^	105.15 ± 8.25 ^bA^
7	Cryptochlorogenic acid	PM	38.97 ± 3.37 ^aB^	38.76 ± 3.62 ^aB^	48.12 ± 4.41 ^aAB^	59.02 ± 5.47 ^aA^	58.66 ± 5.98 ^aA^
		FM	77.42 ± 6.99 ^bB^	101.06 ± 8.83 ^bA^	76.16 ± 6.18 ^bB^	103.66 ± 1.18 ^bA^	103.97 ± 2.99 ^bA^
8	*p*-Hydroxybenzoic acid	PM	2.93 ± 0.21 ^aB^	3.91 ± 0.22 ^aA^	3.70 ± 0.34 ^aA^	1.68 ± 0.11 ^aC^	0.26 ± 0.02 ^aD^
		FM	4.08 ± 0.49 ^bB^	4.78 ± 0.43 ^aAB^	5.42 ± 0.53 ^bA^	0.35 ± 0.02 ^bC^	0.25 ± 0.05 ^aC^
9	3,4-Dihydroxyphenylacetic acid	PM	522.38 ± 19.13 ^aA^	462.38 ± 92.82 ^aA^	529.90 ± 9.62 ^aA^	494.60 ± 17.64 ^aA^	477.02 ± 6.48 ^aA^
		FM	312.20 ± 4.91 ^bB^	380.69 ± 8.76 ^bA^	354.27 ± 17.77 ^bA^	318.83 ± 11.23 ^bB^	177.24 ± 4.55 ^bC^
10	Epicatechin	PM	0.96 ± 0.02 ^aB^	0.62 ± 0.04 ^aC^	0.62 ± 0.06 ^aC^	0.51 ± 0.04 ^aC^	1.54 ± 0.17 ^aA^
		FM	0.52 ± 0.08 ^bBC^	0.42 ± 0.06 ^bD^	0.65 ± 0.07 ^aA^	0.57 ± 0.05 ^aAB^	0.45 ± 0.07 ^bCD^
11	Caffeic acid	PM	0.41 ± 0.03 ^aC^	0.94 ± 0.07 ^aB^	1.10 ± 0.12 ^aB^	1.40 ± 0.16 ^aA^	0.03 ± 0.00 ^aD^
		FM	1.70 ± 0.27 ^bB^	2.67 ± 0.18 ^bA^	2.92 ± 0.24 ^bA^	2.45 ± 0.28 ^bA^	Nd
12	Syringic acid	PM	0.22 ± 0.01 ^aC^	5.46 ± 0.57 ^aA^	2.57 ± 0.22 ^aB^	0.42 ± 0.01 ^aC^	0.60 ± 0.08 ^aC^
		FM	5.00 ± 0.59 ^bB^	2.90 ± 0.21 ^bC^	8.06 ± 0.77 ^bA^	5.63 ± 0.51 ^bB^	0.60 ± 0.01 ^aD^
13	Vanillic acid	PM	0.84 ± 0.09 ^aBC^	0.78 ± 0.05 ^aBC^	1.15 ± 0.17 ^aA^	0.75 ± 0.07 ^aC^	1.02 ± 0.04 ^aAB^
		FM	1.10 ± 0.15 ^aAB^	0.94 ± 0.05 ^bBC^	1.06 ± 0.06 ^aBC^	0.80 ± 0.17 ^aC^	1.35 ± 0.03 ^bA^
14	Benzoic acid	PM	205.28 ± 23.70 ^aD^	1874.22± 115.46 ^aB^	2826.56 ± 1.73 ^aA^	586.49 ± 22.32 ^aC^	94.96 ± 18.70 ^aE^
		FM	895.92 ± 70.05 ^bB^	2920.44 ± 50.39 ^bA^	3118.91 ± 53.83 ^bA^	471.66 ± 18.92 ^bC^	271.28 ± 17.98 ^bD^
15	2-O-Rhamnosylvitexin	PM	0.26 ± 0.06 ^aB^	0.45 ± 0.00 ^aA^	0.34 ± 0.05 ^aB^	0.28 ± 0.02 ^aB^	0.32 ± 0.05 ^aB^
		FM	0.29 ± 0.02 ^aAB^	0.27 ± 0.02 ^bAB^	0.33 ± 0.01 ^aA^	0.25 ± 0.04 ^aB^	0.26 ± 0.02 ^aB^
16	Rutin	PM	4.75 ± 0.41 ^aAB^	5.32 ± 0.53 ^aA^	5.82 ± 0.47 ^aA^	5.14 ± 0.43 ^aA^	4.43 ± 0.57 ^aB^
		FM	5.34 ± 0.53 ^aA^	6.67 ± 0.52 ^bA^	5.98 ± 0.62 ^aA^	6.16 ± 0.61 ^aA^	6.06 ± 0.65 ^bA^
17	*p*-Coumaric acid	PM	2.27 ± 0.28 ^aB^	5.28 ± 0.56 ^aA^	5.68 ± 0.17 ^aA^	1.92 ± 0.18 ^aB^	Nd
		FM	5.01 ± 0.57 ^bC^	6.89 ± 0.56 ^bB^	8.65 ± 0.88 ^bA^	1.35 ± 0.12 ^bD^	Nd
18	Hyperoside	PM	3.19 ± 0.25 ^aA^	3.21 ± 0.33 ^aA^	3.62 ± 0.36 ^aA^	2.89 ± 0.26 ^aA^	1.82 ± 0.19 ^aB^
		FM	2.84 ± 0.22 ^aB^	3.64 ± 0.36 ^aA^	3.40 ± 0.29 ^aA^	2.96 ± 0.25 ^aAB^	2.41 ± 0.14 ^bB^
19	Isoquercetin	PM	2.52 ± 0.09 ^aAB^	2.63 ± 0.12 ^aA^	2.86 ± 0.11 ^aA^	2.37 ± 0.26 ^aB^	1.42 ± 0.10 ^aC^
		FM	2.31 ± 0.27 ^aAB^	2.90 ± 0.21 ^aA^	2.67 ± 0.29 ^aA^	2.37 ± 0.22 ^aAB^	1.90 ± 0.13 ^bB^
20	Ferulic acid	PM	0.71 ± 0.01 ^aB^	0.80 ± 0.07 ^aAB^	0.87 ± 0.03 ^aA^	0.87 ± 0.06 ^aA^	0.88 ± 0.05 ^aA^
		FM	0.34 ± 0.03 ^bC^	0.46 ± 0.01 ^bBC^	0.55 ± 0.06 ^bB^	0.66 ± 0.06 ^bA^	0.35 ± 0.02 ^bC^
21	Isoferulic acid	PM	11.7 ± 1.12 ^aA^	12.63 ± 0.72 ^aA^	14.13 ± 1.38 ^aA^	8.53 ± 0.76 ^aB^	6.99 ± 0.72 ^aB^
		FM	2.08 ± 0.19 ^bC^	2.88 ± 0.26 ^bB^	3.71 ± 0.35 ^bA^	3.99 ± 0.31 ^bA^	3.99 ± 0.32 ^bA^
22	Kaempferol-3-O-rutinoside	PM	0.17 ± 0.03 ^aA^	0.16 ± 0.01 ^aA^	0.20 ± 0.03 ^aA^	0.18 ± 0.02 ^aA^	0.12 ± 0.01 ^aB^
		FM	0.16 ± 0.02 ^aB^	0.24 ± 0.02 ^bA^	0.21 ± 0.01 ^aA^	0.22 ± 0.03 ^aA^	0.18 ± 0.02 ^bB^
23	Quercetin	PM	0.66 ± 0.05 ^aC^	0.76 ± 0.02 ^aBC^	0.80 ± 0.06 ^aAB^	0.92 ± 0.09 ^aA^	0.84 ± 0.05 ^aAB^
		FM	1.55 ± 0.19 ^bB^	1.62 ± 0.13 ^bAB^	1.79 ± 0.14 ^bA^	1.95 ± 0.13 ^bA^	1.91 ± 0.17 ^bA^
24	Naringenin	PM	30.21 ± 3.52 ^aB^	33.81 ± 2.86 ^aB^	37.76 ± 3.52 ^aAB^	44.01 ± 4.34 ^aA^	44.20 ± 4.01 ^aA^
		FM	49.82 ± 4.81 ^bA^	47.00 ± 4.77 ^bAB^	41.40 ± 4.80 ^bB^	45.47 ± 5.59 ^aAB^	53.23 ± 4.08 ^bA^

Nd, not detected; PM, *Prunus mume*; FM, *Fructus mume*. Fermentation broth in different times: F1, 1 h; F3, 3 h; F6, 6 h; F12, 12 h; F24, 24 h. 3-(3,4-dihydroxyphenyl) propionic acid, 3,4-dihydroxyphenylacetic acid and benzoic acid were quantified in *p*-hydroxybenzoic acid equivalents. Data represent mean ± standard deviation (*n* = 3). Different lowercase letters represent a significant difference between PM and FM at same phase (*p* < 0.05). Different capital letters represent a significant difference at different phases (*p* < 0.05).

### 3.5. Correlation Between Phenolics Compounds and Gut Microbiota

**Figure 5:**
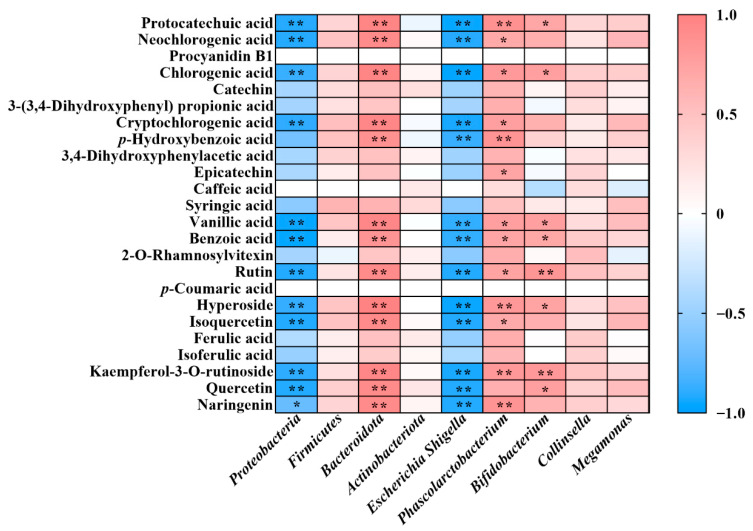
Heatmap of correlation analysis between gut microbiota and individual phenolics. Red squares represent positive correlations, blue squares represent negative correlations and white squares represent no correlation. * *p* < 0.05, ** *p* < 0.01.

The correlation between the content of major phenolic compounds released after 24 h of fermentation and the relative abundance of gut microbiota was examined using the Spearman correlation analysis (Figure 5). The protocatechuic acid, chlorogenic acid, vanillic acid, benzoic acid, rutin, hyperoside and kaempferol-3-O-rutinoside had a significantly negative correlation with *Proteobacteria* and *Escherichia–Shigella* (*p* < 0.01), and a significantly positive relationship with *Bacteroidota* (*p* < 0.01), *Phascolarctobacterium* (*p* < 0.05) and *Bifidobacterium* (*p* < 0.05). The stronger inhibitory activity of FM against *Proteobacteria* and *Escherichia–Shigella* might be attributed to the higher release of chlorogenic acid and benzoic acid in FM compared to PM (*p* < 0.05). Moreover, FM’s greater promotion of beneficial gut microbes such as *Bifidobacterium* and *Phascolarctobacterium* might be related to its higher released content of neochlorogenic acid, cryptochlorogenic acid, chlorogenic acid and benzoic acid relative to PM (*p* < 0.05). These results demonstrated that PM and FM possessed prebiotic potentials, which could be further enhanced after PM-processing (*paozi*).

## 4. Conclusions

The release characteristics and antioxidant activity change of phenolics from PM and FM during in vitro simulated digestion as well as gut microbiota modulation effect were firstly determined and compared in the present study. Both the total polyphenols content released and antioxidant activities of FM were much higher than those of PM during intestinal digestion and colonic fermentation. Importantly, compared with PM, FM demonstrates superior effects on colonic health by increasing the relative abundance of probiotic bacteria such as *Bifidobacterium* to a greater extent and exerting stronger inhibitory effects against *Escherichia–Shigella*. These results provided insights into the digestive properties of polyphenols of PM and FM after oral intake, indicating that FM processing potentially increased PM’s health effect on the colon. The study also provides a theoretical basis for FM used as a traditional medicine for gastrointestinal disorders. However, considering processing-induced changes in non-phenolic components (e.g., polysaccharide degradation, Maillard reaction products during heating), the differential effects of PM vs. FM on regulating the gut microbiota composition could not be ascribed solely to processing-induced alterations in phenolic compounds. Further exploration is required to determine how FM processing-induced changes in non-phenolic components affect the prebiotic potential of PM.

## Data Availability

The original contributions presented in the study are included in the article/Supplementary Material, further inquiries can be directed to the corresponding authors.

## Supplementary Materials

The following supporting information can be downloaded at: https://www.mdpi.com/article/10.3390/foods14234067/s1, Figure S1: The primary and secondary mass spectra of vanillic acid (A) and syringic acid (B); Figure S2: The primary and secondary mass spectra of kaempferol-3-O-rutinoside (A) and 2-O-Rhamnosylvitexin (B); Table S1: Configuration of simulated saliva, gastric and intestinal fluids (SSF, SGF, SIF) and preheat to 37 °C before use; Table S2: Calibration curves used for UPLC-MS/MS quantification of polyphenols.
