# Supplementary material for: Bioaccessibility and Gut Microbiota Modulation of Phenolics in Prunus mume vs. Fructus mume
Source: Foods. 2025 Nov 27;14(23):4067. doi: 10.3390/foods14234067 (PMC12691899; doi:10.3390/foods14234067)
Supplement: Supplementary file 1 [file foods-14-04067-s001.zip › foods-3972653-supplementary.pdf]

Figure captions

Fig. S1. The primary and secondary mass spectra of vanillic acid (A) and syringic acid (B)

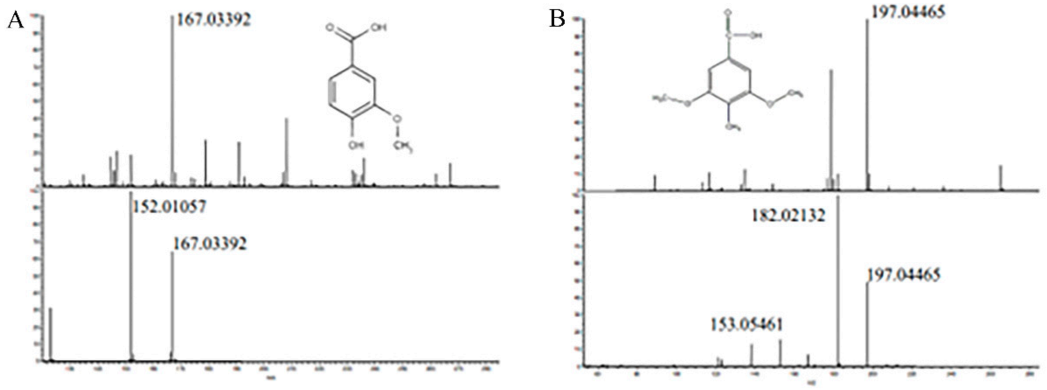

Fig. S2. The primary and secondary mass spectra of kaempferol-3-O-rutinoside (A) and 2-O-Rhamnosylvitexin (B)

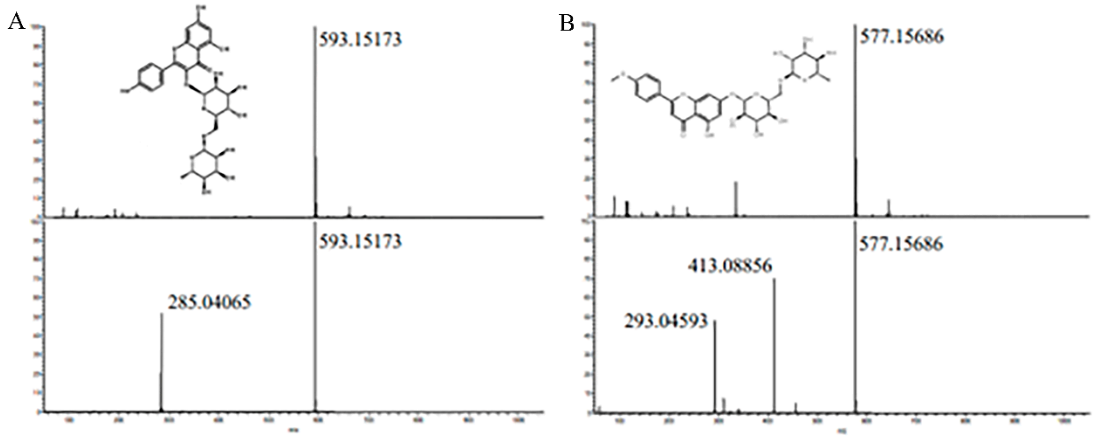

Table S1

Configuration of simulated saliva, gastric and intestinal fluids (SSF, SGF, SIF) and preheat to 37 °C before use.

Reagent	Concentration（g/L)	SSF（mL)	SGF (mL)	SIF（mL)
KCl	37.3	15.1	6.9	6.8
KH_2PO_4	68	3.7	0.9	0.8
$NaHCO_3$	84	6.8	12.5	42.5
NaCl	117	-	11.8	9.6
$MgCl_2(H_2O)_6$	0.5	0.5	0.4	1.1
$(NH_4)_2CO_3$	48	0.06	0.5	-
HCl	6 (M)	0.09	1.3	0.7

Table S2

Calibration curves used for UPLC-MS/MS quantification of polyphenols.

Phenolic compounds	Calibration curves	Correlation coefficients (R^2)	The range of tested concentrations (ng/mL)
Protocatechuic acid	Y = 6.37757e+006+68383.1*X	0.9972	23.976-4986.620
Neochlorogenic acid	Y = -29085.1+32930.4*X	0.9998	1.363-4990.405
Procyanidin B1	Y = 45134+7807.99*X	0.9998	37.072-4992.674
Chlorogenic acid	Y = 202060+32151.4*X	0.9998	30.406-4993.530
Catechin	Y = 2.34287e+006+48709*X	0.9991	6.666-4980.523
Cryptochlorogenic acid	Y = 707951+33947.1*X	0.9996	19.438-4988.313
p-Hydroxybenzoic acid	Y = 4.62199e+006+54280.2*X	0.9979	187.197-4968.528
Epicatechin	Y = 1.72757e+006+57221*X	0.9995	25.312-4988.259
Caffeic acid	Y = 1.55712e+007+167806*X	0.9985	196.343-4954.110
Syringic acid	Y = -333801+8493.29*X	1.0000	33.141-4984.034
Vanillic acid	Y = -428201+4420.47*X	0.9999	220.405-5005.013
2-O-Rhamnosylvitexin	Y = 942076+20620.7*X	0.9989	12.182-4973.838
Rutin	Y = -305408+21668.2*X	1.0000	17.181-5002.292

p-Coumaric acid	Y = 1.08762e+007+111677*X	0.9941	190.307-4970.865
Hyperoside	Y = 237202+34774*X	0.9998	40.717-4993.358
Isoquercetin	Y = 1.17999e+006+28955.4*X	0.9988	11.424-4970.218
Ferulic acid	Y = 2.33102e+006+51082.6*X	0.9990	32.504-4990.759
Isoferulic acid	Y = -463468+3578.82*X	1.0000	199.417-5000.912
Kaempferol-3-O-rutinoside	Y = 1.31022e+006+23196.2*X	0.9978	0.498-4961.502
Quercetin	Y = 9.03669e+006+97953*X	0.9963	163.085-4955.625
Naringenin	Y = -158894+644.172*X	1.0000	497.881-4999.735
